# Combined Use of Serum Uromodulin and eGFR to Estimate Mortality Risk

**DOI:** 10.3389/fmed.2021.723546

**Published:** 2021-09-08

**Authors:** Babak Yazdani, Graciela E. Delgado, Hubert Scharnagl, Bernhard K. Krämer, Heinz Drexel, Winfried März, Jürgen E. Scherberich, Andreas Leiherer, Marcus E. Kleber

**Affiliations:** ^1^Vth Department of Medicine (Nephrology, Hypertensiology, Rheumatology, Endocrinology, Diabetology), Medical Faculty Mannheim, University of Heidelberg, Mannheim, Germany; ^2^Center for Preventive Medicine and Digital Health Baden-Württemberg, Medical Faculty Mannheim, Heidelberg University, Mannheim, Germany; ^3^Clinical Institute of Medical and Chemical Laboratory Diagnostics, Medical University of Graz, Graz, Austria; ^4^European Center for Angioscience, Mannheim, Germany; ^5^Vorarlberg Institute for Vascular Investigation and Treatment at the Academic Teaching Hospital Feldkirch, Feldkirch, Austria; ^6^Private University of the Principality of Liechtenstein, Triesen, Liechtenstein; ^7^Drexel University College of Medicine, Philadelphia, PA, United States; ^8^Division of Angiology, Swiss Cardiovascular Center, University Hospital of Bern, Bern, Switzerland; ^9^Synlab Academy, Synlab Holding Deutschland GmbH, Mannheim, Germany; ^10^Klinikum München-Harlaching, Teaching Hospital of the Ludwig-Maximilians University, Munich, Germany; ^11^KfH-München Süd, Munich, Germany; ^12^Medical Central Laboratories, Feldkirch, Austria; ^13^SYNLAB MVZ Humangenetik Mannheim, Mannheim, Germany

**Keywords:** EGFR, mortality, chronic kidney disease, Tamm-Horsfall protein, uromodulin

## Abstract

Serum uromodulin (sUmod) shows a strong direct correlation with eGFR in patients with impaired kidney function and an inverse association with mortality. However, there are patients in whom only one of both markers is decreased. Therefore, we aimed to investigate the effect of marker discordance on mortality risk. sUmod and eGFR were available in 3,057 participants of the Ludwigshafen Risk and Cardiovascular Health study and 529 participants of the VIVIT study. Both studies are monocentric prospective studies of patients that had been referred for coronary angiography. Participants were categorized into four groups according to the median values of sUmod (LURIC: 146 ng/ml, VIVIT: 156) and eGFR (LURIC: 84 ml/min/1.73 m^2^, VIVIT: 87). In 945 LURIC participants both markers were high (UHGH), in 935 both were low (ULGL), in 589 only eGFR (UHGL), and in 582 only sUmod (ULGH) was low. After balancing the groups for cardiovascular risk factors, hazard ratios (95%CI) for all-cause mortality as compared to UHGH were 2.03 (1.63–2.52), 1.43 (1.13–1.81), and 1.32 (1.03–1.69) for ULGL, UHGL, and ULGH, respectively. In VIVIT, HRs were 3.12 (1.38–7.08), 2.38 (1.01–5.61), and 2.06 (0.81–5.22). Adding uromodulin to risk prediction models that already included eGFR as a covariate slightly increased the Harrell's C and significantly improved the AUC in LURIC. In UHGL patients, hypertension, heart failure and upregulation of the renin-angiotensin-aldosterone-system seem to be the driving forces of disease development, whereas in ULGH patients metabolic disturbances might be key drivers of increased mortality. In conclusion, SUmod/eGFR subgroups mirror distinct metabolic and clinical patterns. Assessing sUmod additionally to creatinine or cystatin C has the potential to allow a more precise risk modeling and might improve risk stratification.

## Introduction

Uromodulin is the most abundant protein in mammalian urine and is also secreted into the blood in small amounts (sUmod). Results from genome-wide association studies linking genetic variation at the *UMOD* locus with estimated glomerular filtration rate (eGFR) (1), chronic kidney disease (CKD) (1–3), arterial hypertension (4) and diabetic nephropathy (5), as well as the recent development of immunoassays to reliably measure sUmod have renewed the scientific interest in this protein. Polymorphisms in the *UMOD* gene explain ~25% of the 4% variability in eGFR that can be explained by genetic factors so far (6). Expression and secretion of uromodulin are regulated by an intricate network of transcription factors (7). Studies in mice and analyses of genetic variants of uromodulin in humans have shown important roles of nuclear factor 1-beta (HNF1b) and a glucocorticoid response element that is disrupted by the presence of the rare allele of a common uromodulin SNP (8). *In silico* analyses also suggested a possible role of a number of other transcription factors such as GATA3, SP1, SP3, TP53, POU2F1, RARB, RARA, RXRA, SMAD3, RUNX2, and KLF4 (7).

In the case of pathophysiological conditions they act together to rapidly modulate Umod concentration. Due to its immunomodulatory and anti-inflammatory properties, sUmod concentrations might be actively increased in the setting of systemic illnesses to reduce systemic inflammation (9, 10). This might partly explain the consistently observed inverse association with cardiovascular risk (11–13).

A report from the SPRINT trial showed baseline urinary uromodulin to be associated with the primary endpoint of cardiovascular events in patients with an eGFR <60 ml/min/1.73 m^2^, independently from eGFR and albuminuria (14). We recently investigated the association of sUmod with mortality in the Ludwigshafen Risk and Cardiovascular Health study (LURIC) and the VIVIT study and found sUmod to be an independent predictor for mortality, even in models adjusted for eGFR in patients with median to high cardiovascular risk (11, 12). Recently, this association has also been confirmed in a population-based study (15). While sUmod showed a strong direct correlation with eGFR there were subgroups of patients in which only one of these markers was decreased. Therefore, we aimed to examine the impact of eGFR/uromodulin discordance on the individual mortality risk and further sought to determine the clinical, biochemical, and genetic characteristics of these subgroups.

## Methods

### Subjects

The LURIC study enrolled 3,316 individuals between 1997 and 2000 at the Ludwigshafen Heart Center in South-West Germany (16) and the VIVIT study 1,048 individuals between 2005 and 2008 at the Landeskrankenhaus Feldkirch in the westernmost province of Austria (17). All participants were of European ancestry, and were referred for elective coronary angiography for the evaluation of established or suspected stable CAD. Patients undergoing coronary angiography for other reasons were not enrolled. The ethics committee of the “Landesärztekammer Rheinland-Pfalz” [LURIC, #837.255.97(1394)] and of “Vorarlberg” (VIVIT, EK-2-2013/0008) approved both studies. Both studies were conducted in accordance with the “Declaration of Helsinki.” Informed written consent was obtained from all participants. For 3,051 study participants of LURIC and 529 of VIVIT both sUmod and eGFR were available and those were used for further analyses. sUmod and eGFR were measured and calculated using the same blood samples. Information on vital status was obtained from local registries. Death certificates, medical records of local hospitals, and autopsy data were reviewed independently by two experienced clinicians who were blinded to patient characteristics and who classified the causes of death. 917 (30.1%) LURIC participants died during a median follow-up of 9.9 years (8.57–10.7) and 93 VIVIT participants during a median follow-up of 7.3 years (7.0–7.6). Fasting blood samples were obtained by venipuncture at study entry. A summary of analytic methods has been reported previously (16, 18) and detailed information regarding the laboratory measurements, and clinical definitions is provided in the Supplementary Material.

### Statistical Analyses

Study participants were categorized into tertiles of sUmod/eGFR or into four groups according to the median values of sUmod (146 ng/ml in LURIC and 156 ng/ml in VIVIT) and eGFR (84 ml/min/1.73 m^2^ in LURIC and 87 in VIVIT). In LURIC, also clinical cutoff criteria were applied to create the groups using cutpoints of 150 ng/ml for sUmod and 60 ml/min/1.73 m^2^ for eGFR. Continuous data are presented as the mean and standard deviation (SD) when normally distributed or as the median and 25th and 75th percentile for non-normally distributed variables. Categorical data are presented as percentages. Statistical differences between groups and continuous variables were determined using ANOVA. Non-normally distributed variables were log-transformed before entering analysis. Tukey's honest significant difference test was used to investigate differences between individual groups using family-wise correction for multiple testing. The chi-square test was used for categorical variables and differences between individual groups were examined using the “chisq.post.hoc” function implemented in the R package “fifer” v1.1 using the false-discovery-rate method for multiple testing correction.

Survival curves for the different groups were calculated by Kaplan-Meier analysis using the R package “survminer” v0.4.3. We also adjusted the distribution of possible confounders by inverse probability weighting, thereby balancing the subgroups for the confounding variables. A weighted Cox model was calculated and we report the result of the robust score test as implemented in the coxph function in R that corresponds to a log-rank test corrected for weighting. The proportional hazard assumption was checked by examination of scaled Schoenfeld residuals. Harrell's C was calculated using the R package hmisc v4.4-1, ROC curves were calculated and compared using the method of Delong as implemented in the R package pROC 1.16.2.

All tests were two-sided and a *P*-value < 0.05 was considered statistically significant. All analyses were carried out using R v4.0.3 [(19) R: A language and environment for statistical computing] (http://www.r-project.org).

## Results

### Association of eGFR and sUmod With Mortality

Information on both sUmod and eGFR CKD-EPI_creat−cys_ was available for 3,051 participants of the LURIC study and 529 participants of VIVIT. Plots showing the correlation between both biomarkers are shown in Supplementary Figure 1.

**Figure 1:**
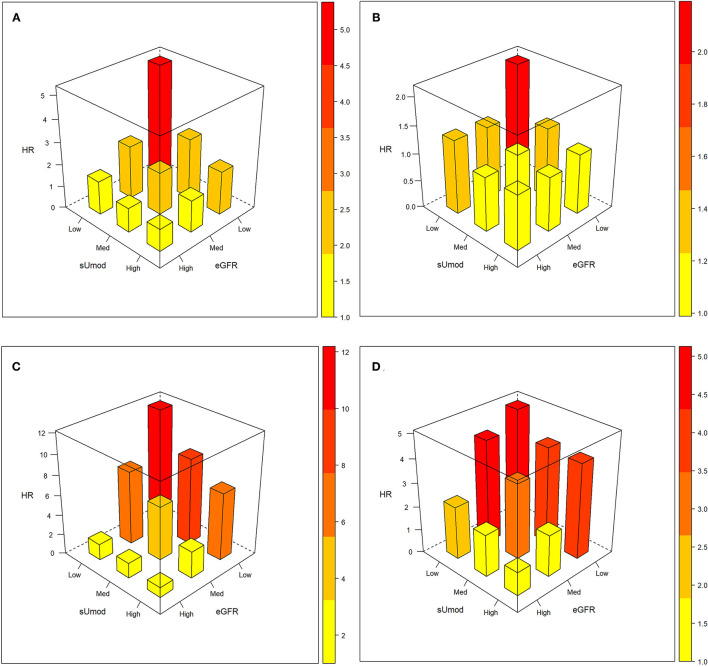
Interaction between sUmod and eGFR with regard to mortality risk. Hazard ratios unadjusted **(A,C)** and with adjustment for age, and with additional adjustment for age, sex, BMI, LDL-C, HDL-C, hypertension, diabetes mellitus, CAD, and smoking **(B,D)**. Upper panel LURIC: (sUmod: ≤118 ng/ml, 119–178 ng/ml, ≥179 ng/ml; eGFR: ≤75 ml/min/1.73 m^2^, 76–92 ml/min/1.73 m^2^, ≥93 ml/min/1.73 m^2^), lower panel VIVIT (sUmod: ≤125 ng/ml, 126–190 ng/ml, ≥191 ng/ml; eGFR: ≤78 ml/min/1.73 m^2^, 78–96 ml/min/1.73 m^2^, ≥96 ml/min/1.73 m^2^).

We stratified our patient cohorts according to tertiles of sUmod and eGFR and calculated hazard ratios (HR) for all-cause mortality for the different combinations (Figure 1; Table 1). As compared to the reference group with both sUmod and eGFR in the highest tertile, the group with both markers in the lowest tertile had a HR of 5.38 (4.18–6.94) in the unadjusted analysis and a HR of 2.19 (1.65–2.91) after adjustment for age, sex, BMI, LDL-C, HDL-C, coronary artery disease (CAD), hypertension, diabetes mellitus (DM), and smoking in LURIC. In VIVIT, the HRs were 12.20 (3.73–39.85) and 5.13 (1.44–18.28) for the unadjusted and the adjusted analysis, respectively. Within each eGFR category, the mortality risk increased with lower sUmod. Vice versa, within each sUmod group the mortality risk increased with lower eGFR.

**Table 1:** All-cause mortality according to sUmod and eGFR.

		**Unadjusted**		**Adjusted**	
**sUmod**	**eGFR**	**HR (95% CI)**	***P***	**HR (95% CI)**	***P***
High	High			1	
High	Medium	1.43 (1.04–1.97)	0.027	0.99 (0.71–1.37)	0.94
		2.68 (0.67–10.73)	0.163	1.77 (0.43–7.25)	0.428
High	Low	1.96 (1.36–2.83)	<0.001	1.10 (0.75–1.61)	0.636
		6.81 (1.76–26.34)	0.005	4.06 (1.00–16.47)	0.05
Medium	High	1.09 (0.77–1.53)	0.635	0.99 (0.70–1.40)	0.956
		1.52 (0.31–7.55)	0.606	1.78 (0.36–8.87)	0.48
Medium	Medium	1.91 (1.41–2.59)	<0.001	1.09 (0.80–1.49)	0.587
		5.48 (1.51–19.91)	0.01	3.23 (0.86–12.15)	0.082
Medium	Low	2.72 (2.04–3.64)	<0.001	1.29 (0.95–1.76)	0.106
		8.72 (2.56–29.76)	0.001	4.10 (1.13–14.92)	0.032
Low	High	1.51 (1.05–2.18)	0.28	1.35 (0.93–1.95)	0.11
		1.64 (0.27–9.79)	0.59	2.24 (0.37–13.47)	0.377
Low	Medium	2.40 (1.77–3.25)	<0.001	1.31 (0.96–1.80)	0.088
		7.45 (2.12–26.14)	0.002	4.41 (1.21–16.00)	0.024
Low	Low	5.38 (4.12–6.94)	<0.001	2.19 (1.65–2.91)	<0.001
		12.20 (3.73–39.85)	<0.001	5.13 (1.44–18.28)	0.012

*Unadjusted and adjusted (for age, sex, BMI, LDL-C, HDL-C, smoking, hypertension, CAD, and DM) HRs are summarized for LURIC (upper rows) and VIVIT (lower rows). Green, marker is high; yellow, marker is at medium level; red; marker is low*.

### Definition of eGFR/sUmod Subgroups

To further examine the groups with discordant sUmod and eGFR, we stratified our cohort into four groups according to the median sUmod and eGFR values. In LURIC, the median value of sUmod was 146 ng/ml, the median value of eGFR was 84 ml/min/1.72 m^2^. In 945 LURIC study participants both markers were above the respective thresholds (UHGH), in 935 both markers were low (ULGL), in 589 only eGFR (UHGL) and in 582 only sUmod (ULGH) was low.

In VIVIT, the median value of sUmod was 156 ng/ml, the median value of eGFR was 87 ml/min/1.72 m^2^. The numbers for the different subgroups were 160, 105, 105, and 160 for UHGH, ULGL, UHGL, and ULGH, respectively.

### sUmod/eGFR Subgroups and Mortality

**Figure 2:**
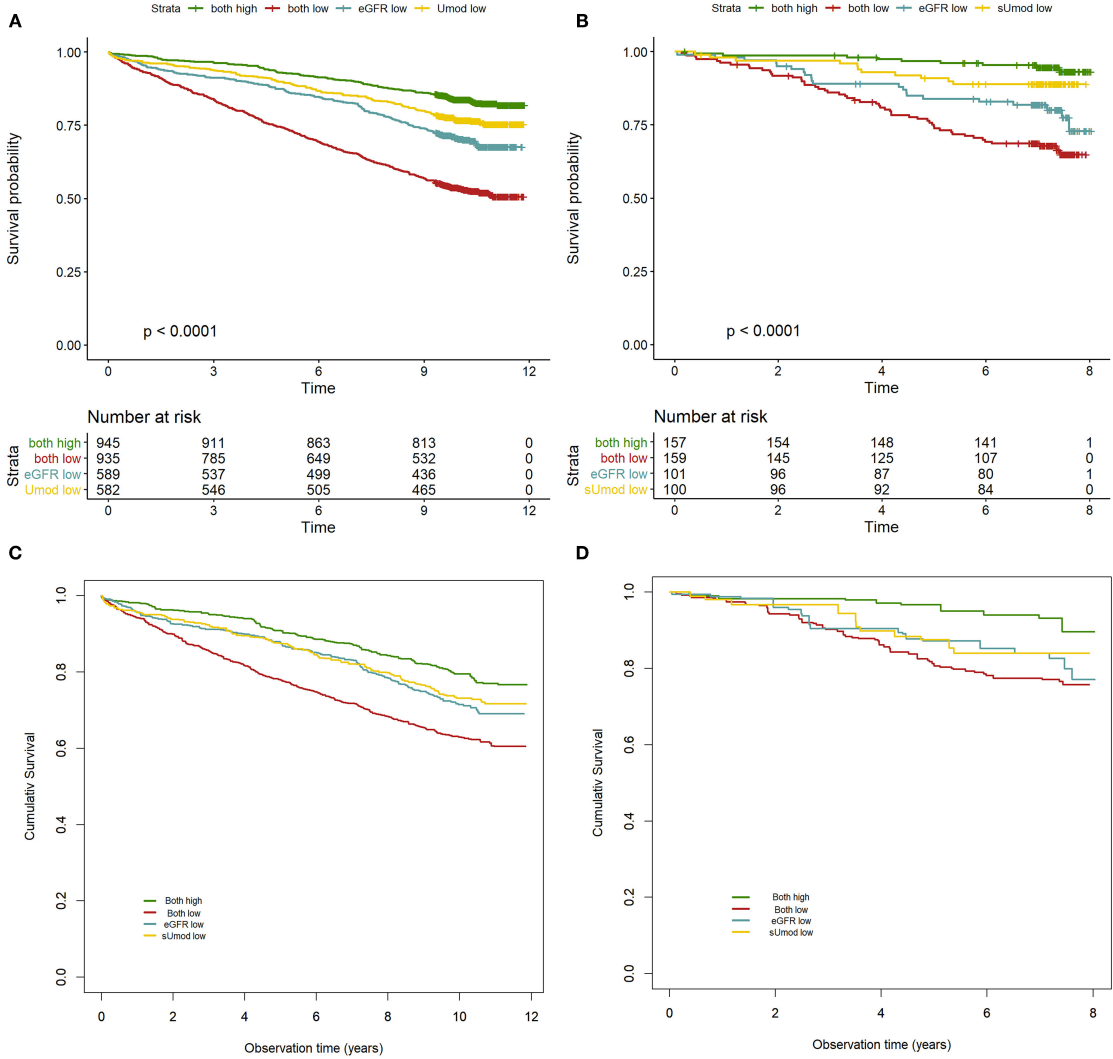
Survival according to eGFR/sUmod groups. Kaplan-Meier survival curves for LURIC and VIVIT **(A,B)** and adjusted survival curves with eGFR/sUmod groups balanced for age, sex, BMI, LDL-C, HDL-C, smoking, hypertension, CAD, and DM by inverse variance weighting for LURIC and VIVIT **(C,D)**. The *P*-value of the robust score test was <0.001 for LURIC and VIVIT.

Kaplan-Meier analysis revealed a higher mortality risk for UHGL as compared to ULGH (Figures 2A,B). As expected, ULGL showed the highest mortality. Adjusted survival curves are shown in Figures 2C,D. The distribution of confounding variables (age, sex, BMI, arterial hypertension, CAD, DM, smoking, LDL-C, and HDL-C) in the four groups was balanced by inverse variance weighting. Resulting HR as compared to UHGH were 2.03 (1.63–2.52), 1.43 (1.13–1.81), and 1.32 (1.03–1.69) for ULGL, UHGL, and ULGH in LURIC, respectively. In the VIVIT cohort, HR as compared to UHGH were 3.12 (1.38–7.08), 2.38 (1.01–5.61), and 2.06 (0.81–5.22) for ULGL, UHGL, and ULGH, respectively. Results for cardiovascular mortality were similar (Supplementary Figure 2).

We also defined the sUmod/eGFR groups in LURIC according to clinical cutoff criteria: As threshold we chose 60 ml/min/1.73 m^2^ for eGFR (to identify those individuals with CKD) and 150 ng/ml for sUmod [approximately below this concentration the mortality risk increases steeply (11)]. The resulting four groups differed substantially in size, especially UHGL was small with only 60 participants, but results were similar (Supplementary Figure 3; Supplementary Table 1). We also calculated a Cox regression model including an interaction term for sUmod and eGFR and this interaction term was highly significant (Supplementary Table 1).

### Performance of Uromodulin in Risk Prediction Models

We investigated whether the addition of uromodulin to risk prediction models including eGFR would improve risk prediction. To answer this question, we examined two models: one including age, sex, BMI, CAD, DM, hypertension, smoking, LDL-C, HDL-C, and eGFR and a second model including all covariates and additionally sUmod (Table 2). The inclusion of uromodulin slightly increased the Harrell's C and the conventional AUC in both models. The improvement in AUC was statistically significant only in the LURIC cohort. However, a trend toward an improved risk prediction could also be observed in the VIVIT cohort.

**Table 2:** Risk prediction models for all-cause mortality with and without inclusion of serum uromodulin.

	**Harrells C**	**AUC (95% CI)**	***P*** *
Age + sex + eGFR	0.709	0.745 (0.726–0.764)	
	0.748	0.778 (0.729–0.827)	
Age + sex + eGFR + sUmod	0.716	0.752 (0.733–0.771)	0.010
	0.754	0.784 (0.737–0.832)	0.366
Base model	0.718	0.771 (0.753–0.788)	
	0.767	0.799 (0.751–0.847)	
Base model + sUmod	0.722	0.774 (0.757–0.792)	0.043
	0.775	0.808 (0.762–0.854)	0.155

* *Model including uromodulin vs. preceding model; base model includes age, sex, BMI, CAD, diabetes mellitus, hypertension, smoking, LDL-C, HDL-C, and eGFR. Upper rows: LURIC, lower rows VIVIT*.

### Characterization of sUmod/eGFR Groups

There were highly significant differences in clinical and biochemical markers between the different sUmod/eGFR groups. In LURIC, comparing UHGH with the groups that had only one marker decreased we observed significantly lower albumin and higher fasting glucose and fatty liver index only in ULGH but not in UHGL (Table 3). Contrary, magnesium was only significantly higher in UHGL but not in ULGH, as compared to UHGH. In VIVIT, results were similar for those variables that were available (Table 4).

**Table 3:** Characteristics of LURIC study participants according to serum uromodulin (<146 or ≥146 ng/mL) and eGFR (<84 or ≥84 mL/min/1.73 m^2^) groups.

**Variable**	**Both high**	**Both low**	**eGFR low**	**sUmod low**	$P_{\text{ANOVA}}^{*}$	***P_post-hoc_***		
	**UHGH**	**ULGL**	**UHGL**	**ULGH**		**UHGH vs. UHGL**	**UHGH vs. ULGH**	**UHGL vs. ULGH**
Age (years)	57(9.76)	68.2(8.69)	67(8.03)	58.7(10.3)	<0.001	<0.001	<0.001	0.003
Male sex (%)	74.6	65.8	57.7	80.4	<0.001	<0.001	<0.001	0.009
BMI (kg/m^2^)	27(3.84)	27.7(4.13)	27.7(4.35)	27.7(4.12)	<0.001	1	0.011	0.007
LDL-C (mg/dl)	119(34.8)	113(34.3)	117(34.7)	116(32.4)	0.001	0.88	0.691	0.221
HDL-C (mg/dl)	40(10.9)	36.8(10.5)	39.9(10.9)	38.3(10.8)	<0.001	0.055	1	0.022
TG (mg/dl)	139(103–188)	154(116–208)	144(106–195)	151(113–215)	<0.001	0.024	0.746	<0.001
systolic BP (mmHg)	137(22.2)	145(24.8)	143(23.8)	140(22.1)	<0.001	0.032	<0.001	0.102
diastolic BP (mmHg)	81.1(11.1)	80.6(11.7)	81.5(11.8)	81.1(10.8)	0.482	0.948	0.935	1
Magnesium (mmol/l)	0.847(0.091)	0.862(0.103)	0.859(0.0945)	0.834(0.0879)	<0.001	<0.001	0.082	0.042
Fasting glucose (mg/dl)	99.7(92.1–110)	105(95.2–126)	102(93.8–117)	104(94.7–122)	<0.001	0.272	0.002	<0.001
hsCRP (mg/l)	2.28(1–6.33)	5.25(2.12–10.7)	3.03(1.45–8.3)	3.06(1.22–7.69)	<0.001	0.619	<0.001	0.006
NT-proBNP (ng/ml)	160(68–420)	679(248–1,860)	375(147–1,010)	175(76–486)	<0.001	<0.001	<0.001	0.289
Renin (pg/ml)	17(9–34)	23.5(12–59)	18(9–41)	18(10–35)	<0.001	0.931	0.227	0.61
Angiotensin II (ng/L)	20(12–35)	20(13–34)	20(13–35)	19(12–34)	0.152	0.813	0.45	0.965
Noradrenalin (ng/l)	276(199–380)	339(232–500)	334(233–477)	281(199–394)	<0.001	<0.001	<0.001	0.999
Albumin (g/dl)	4.48(0.535)	4.28(0.553)	4.39(0.524)	4.38(0.573)	<0.001	0.999	0.008	0.007
GOT (U/l)	11.8(7.49)	11.7(7.4)	12.5(9.94)	11.7(6.77)	0.251	0.276	0.338	0.988
FV (U/dl)	112(20.9)	113(23)	114(20.1)	115(21.9)	0.368	0.895	0.829	0.288
Fatty liver index	47.4(25.2–71.4)	58(36.6–77.2)	53.9(31.5–74.3)	55.3(32.1–79.1)	<0.001	0.476	0.006	<0.001
eGFR (ml/min/1.73 m^2^)	98.3(9.42)	62(16.2)	72(9.75)	96.5(9.23)	<0.001	<0.001	<0.001	0.022
Uromodulin (ng/ml)	222(62.6)	95.4(31.4)	202(52.4)	107(28.1)	<0.001	<0.001	<0.001	<0.001
CystatinC (mg/l)	0.8(0.74–0.86)	1.12(1–1.37)	1.01(0.93–1.11)	0.83(0.76–0.89)	<0.001	<0.001	<0.001	0.031
Diabetes mellitus (%)	28.1	51.9	41.1	39	<0.001	0.475	<0.001	<0.001
Coronary artery disease (%)	71.7	83.6	76.1	80.2	<0.001	0.097	0.097	0.001
Heart failure (%)	21.8	47.5	35.1	26.1	<0.001	0.001	<0.001	0.054
Hypertension (%)	62.4	81	77.8	70.3	<0.001	0.005	0	0.003
Smoking (active/ex/never, %)	28.4/38.9/32.7	17.6/45.9/36.5	15.3/37.7/47	31.3/40.5/28.2	<0.001	<0.001	<0.001	0.161

*P-value adjusted for multiple testing using the false-discovery-rate method; GOT, glutamate oxalacetate transaminase; FV, factor V; NT-proBNP, N-terminal pro brain natriuretic peptide; TG, triglycerides.

**Table 4:** Characteristics of VIVIT study participants according to serum uromodulin (<156 or156 ≥ ng/mL) and eGFR (<87 or ≥87 mL/min/1.73 m^2^) groups.

**Variable**	**Both high**	**Both low**	**sUmod high, eGFR low**	**sUmod low, eGFR high**	***P_ANOVA_***	***P_post-hoc_***		
	**UHGH**	**ULGL**	**UHGL**	**ULGH**		**UHGH vs. UHGL**	**UHGH vs. ULGH**	**UHGL vs. ULGH**
*N*	160	160	104	105				
Age (years)	59(10)	72(8.4)	69(8.4)	59(11)	<0.001	<0.001	1	<0.001
Male sex (%)	71	63	49	72	0.001	0.001	0.998	0.002
BMI (kg/m^2^)	28(4)	28(5)	27(4)	28(4)	0.125	0.301	0.976	0.203
LDL-C (mg/dl)	134(38.6)	126(42.1)	131(38.5)	129(38.4)	0.426	0.951	0.763	0.978
HDL-C (mg/dl)	58(18)	58(16)	61(16)	57(15)	0.251	0.421	0.945	0.232
TG (mg/dl)	123(86.3–175)	112(81.3–148)	107(78.0–145)	112(85–175)	0.068	0.080	0.996	0.199
systolic BP (mmHg)	135(17.2)	139(18.7)	139(16.9)	131(18.3)	0.006	0.447	0.345	0.023
diastolic BP (mmHg)	82(10.3)	83(10.8)	83(9.2)	82(9.8)	0.986	0.997	0.999	0.991
Fasting glucose (mg/dl)	98(90.0–1,113)	104(92.0–122)	97(87.0–112)	101(93.5–116)	0.049	0.740	0.750	0.260
CRP (mg/dl)	0.21(0.10–0.39)	0.31(0.16–0.57)	0.19(0.10–0.39)	0.22(0.10–0.47)	0.002	0.961	0.986	0.873
NT-proBNP (ng/ml)	124(64.8–265)	573(160–1,653)	255(172–1,053)	85.5(40.0–276)	<0.001	<0.001	0.716	<0.001
Total protein (g/dl)	7.25(0.50)	7.27(0.47)	7.24(0.46)	7.34(0.36)	0.346	0.995	0.401	0.361
GOT (U/l)	25(21–32)	25(21–29)	25(21–30)	24(21–30)	0.219	1.000	0.766	0.837
Fatty liver index	56.3(25.8)	57.3(26.3)	51.0(26.8)	59.3(27.5)	0.154	0.433	0.814	0.134
eGFR (ml/min/1.73 m^2^)	102(9.0)	65(16)	75(9)	101(9)	<0.001	<0.001	0.937	<0.001
Uromodulin (ng/ml)	229(68.8)	102(32.0)	215(56.5)	114(28.1)	<0.001	0.126	<0.001	<0.001
Cystatin C (mg/l)	0.78(0.69–0.83)	1.10(0.98–1.26)	0.98(0.91–1.06)	0.78(0.72–0.84)	<0.001	<0.001	0.780	<0.001
Diabetes mellitus (%)	23	34	22	31	0.051	1	0.484	0.527
Coronary artery disease (%)	80	82	76	80	0.682	0.986	0.873	0.748
Hypertension (%)	84	92	89	84	0.092	0.527	1	0.613
Smoking (active/ex/never, %)	23/45/32	11/46/43	14/36/50	21/46/33	0.024	0.259	0.968	0.594

Comparing only UHGL and ULGH, we found that participants in UHGL were older and more often female compared to ULGH. They suffered more often from arterial hypertension and had a tendency toward higher rates of heart failure. Study participants in the groups with low sUmod (ULGL+ULGH) had a higher fatty liver index as compared to the groups with high uromodulin (UHGH+UHGL) but the difference was only significant in LURIC. Looking at differences between UHGL and ULGH groups defined by the clinical cutoff criteria, again significant associations (*p* < 0.001) were found for the variables age, male gender, heart failure, NTproBNP, and cystatin C (Supplementary Table 2). For VIVIT we could not use these criteria due to the low number of samples in the UHGL group (*N* = 8).

## Discussion

### Main Findings

Despite a highly significant, direct association of sUmod and eGFR we detected subgroups of patients in whom only one of these biomarkers was decreased. Regarding all-cause and cardiovascular mortality, there was an almost linear increase in mortality risk from UHGH < ULGH < UHGL < ULGL. These findings suggest, that uromodulin modulates mortality risk in patients with both impaired and normal renal function in the way that high uromodulin is associated with higher patient survival. Conversely, in patients with both high and low uromodulin, low eGFR is each time associated with inferior patient survival. When comparing the impact of low eGFR and low uromodulin on all-cause and cardiovascular mortality, the impact of low eGFR appeared to be somewhat stronger than the effect of uromodulin. Adding sUmod to risk prediction models already including eGFR significantly improved risk prediction in LURIC. Furthermore, when comparing UHGL with ULGH, we found significant differences in age, sex, NT-proBNP, and prevalent heart failure.

So far, uromodulin is the only known kidney-specific protein (that can also be measured in the blood) and it has been suggested to serve as a biomarker for nephron mass (20) or the renal functional reserve. Recently, Pruijm et al. reported a strong correlation between 24-h uromodulin excretion with kidney length and volume determined by renal ultrasonography as well as an association with markers of tubular function in a population-based cohort (21) and conclude that uromodulin “may reflect tubule activity in the general population.” Indeed, mutations in the uromodulin gene give rise to autosomal dominant tubulointerstitial kidney disease uromodulin-related (ADTKD-UMOD) and reduced uromodulin excretion has been reported for a number of renal diseases, e.g., glomerulonephritis, diabetic nephropathy, or IgA nephropathy (6, 22–25). In IgA nephropathy, uromodulin was shown to be associated with interstitial fibrosis/tubular atrophy and to contribute to eGFR decline (24). A report from the SPRINT trial showed that lower uromodulin in urine was associated with incident acute kidney injury, independent of eGFR and albuminuria (26). On the other hand, in healthy kidney donors no correlation between serum uromodulin and eGFR has been observed (27). In addition, large GWAS metaanalyses have identified polymorphisms in the UMOD promoter that are associated with a lower concentration of uromodulin but higher eGFR (1), a lower risk for CKD, lower blood pressure, a lower risk of hypertension (4), and left atrial remodeling (28). This is explained by the fact that uromodulin increases the activity of ion channels like the NaKCC2 ion transporter or ROMK in the TAL and thereby enhances salt uptake, which then leads to higher blood pressure and increased cardiorenal risk.

It has been suggested that the link between uromodulin and NaKCC2 is mediated through the regulation of intracellular chloride levels and modulation of the chloride sensitive WNK-SPAK/OSR1 pathway (29, 30), which leads to an increased phosphorylation of NKCC2. The mechanism by which uromodulin modulates SPAK/OSR1 activity could involve potentialization of with-no-lysine kinase (WNK) activity, as previously suggested (29).

NKCC2 and uromodulin are distributed in close spatial vicinity on the surface of TAL cells and both share the same lipid raft localization (31) and so it has been proposed that uromodulin might act as a scaffold for WNK-SPAK/OSR1-dependent activation of NKCC2. Recently it has been demonstrated that the activity of this pathway is increased in the kidneys of mice lacking hepsin, a transmembrane serine protease, which is critical for the luminal release of uromodulin (32). The authors could also show that this goes along with an increased phosphorylation of NKCC2.

It has been shown that Umod knockout mice are resistant to salt-induced changes in blood pressure and a transcriptome study reported that Umod is necessary for the upregulation of heat shock transcripts and the transcripts of seven electrolyte transporters in response to salt stress (33). Further, it has recently been reported that uromodulin also plays a role in the reabsorption of NaCl and the fine-tuning of urinary calcium and magnesium excretion in the distal convoluted tubule (34, 35). In our study, the concentration of magnesium was significantly higher in the groups with low sUmod in LURIC. Another explanation for the seemingly paradoxical association of Umod promoter variants with lower uromodulin but higher eGFR proposes that the effect of these SNPs on kidney risk is independent of Umod expression but due to effects on the expression of neighboring or distal genes that are involved in kidney disease (10).

In large proportions of our cohorts, we observed a discordant behavior between sUmod and eGFR (38% in LURIC, 40% in VIVIT). Regarding ULGH it has been shown that lower concentrations of sUmod may be a novel marker to identify early kidney function loss even when serum creatinine values are still within a normal range (36). The synthesis of Umod per functioning remaining nephron unit is increased in patients with CKD, but may not compensate the overall loss of renal parenchyma (37). In a similar manner, quantitative enzyme and immunohistological analyses of kidney tissue sections from patients with endstage CKD disclosed upregulation of various proteins in single “resistant” hypertrophic and hypermetabolic nephrons (38). Although uromodulin synthesis and intracellular protein transfer might not be disturbed in these surviving nephrons, preinjured epithelia of the thick ascending limb of Henle (TAL) segment should downmodulate uromodulin secretion at a very early stage. This thesis is supported by the observation that sUmod levels decline in early CKD where creatinine and cystatin c concentrations still remain unchanged (39).

This proposed state of early renal damage, with a mean eGFR difference of 25 ml/min/1.73 m^2^ in LURIC and 26 ml/min/1.73 m^2^ in VIVIT, may partly explain the higher mortality in ULGH vs. UHGH patients groups. ULGH patients might presumably also display a more rapid CKD progression; however this has not been studied in LURIC. In VIVIT, serum creatinine has been measured at study baseline and at a 4-year follow-up. Creatinine increased stronger in the ULGH group (0.78–0.83; paired *t*-test *p* = 0.002) as compared to the UHGL group (0.89–0.92; *p* = 0.159).

Regarding the survival advantage of the ULGH group compared to the ULGL group, the main cause probably is renal function, that is on average at an eGFR of 62 mL/min/1.73 m^2^ and is considerably higher as compared to ULGL, despite an early, pre-clinical renal damage [when compared to UHGH].

Importantly, rates of hypertension and heart failure are higher in UHGL than in ULGH in LURIC, suggesting that sodium and fluid overload occur that relate to high sUmod and cannot be balanced by a largely preserved renal function such as in the ULGH patient group.

In VIVIT, there was no significant difference in the rates of hypertension between both groups. Information on heart failure was not available in this cohort but the concentration of NT-proBNP was almost three times higher in the ULGH group.

Conversely, when analyzing the higher mortality in UHGL vs. UHGH patients groups, a difference in mortality that is exceeding the difference between ULGH vs. UHGH by far, the obvious culprit is impaired renal function, that associates with higher rates of arterial hypertension, heart failure, and DM. The survival advantage when comparing UHGL with ULGL may be explained by protective, i.e., antiinflammatory and immunomodulatory effects of normal sUmod levels and by a somewhat better renal function. The difference in eGFR between both groups was 10 ml/min/1.73 m^2^ in favor of the UHGL patient group.

Applying a stricter clinical cutoff of 60 ml/min/1.73 m^2^ for eGFR in LURIC, we found significant differences between UHGL and ULGH regarding a number of vasoactive biomarkers that were not apparent in the analyses using the median groups (Supplementary Table 1). NT-proBNP and angiotensin II were significantly higher in UHGL as compared to ULGH. It is important to notice that the percentage of patients suffering from heart failure and hypertension was higher in UHGL, possibly due to a sUmod mediated enhanced tubular activation of the NKCL-cotransporter. It is tempting to speculate that elevated central venous pressure in heart failure might promote renal congestion (40). A reduced kidney perfusion pressure, associated with potential medullary hypoxia, may lead to an activation of the sympathetic nervous system and an upregulation of the renin-angiotensin-aldosterone (RAAS) system, which helps to explain the elevated concentrations of renin, angiotensin II and noradrenaline in UHGL. Increased concentrations of angiotensin-II and catecholamines consecutively promote glomerular arteriolar vasoconstriction, thereby decreasing renal plasma flow and ultimately eGFR (41). On the other hand, patients in the ULGH group showed higher triglycerides, lower mean LDL particle radius, higher fatty liver index and a higher prevalence of CAD as compared to UHGL.

Of note, a recent study found that sUmod function might be impaired by carbamylation in the setting of CKD (42) and that could also partly account for the higher mortality in the UHGL group as compared to ULGH.

### Strengths and Limitations

All LURIC and VIVIT Study participants were of European origin, therefore our findings may not be applicable to other ethnicities. Both studies recruited participants that had been referred for coronary angiography. Our results may therefore not be generalizable to a healthy population. Uromodulin was only measured once in baseline samples. Urine samples were not available to assess urinary uromodulin and eGFR was only calculated once at baseline in LURIC.

The major strengths of our cohorts are, however, the precise clinical and metabolic characterization of the participants, including the availability of coronary angiograms, their cross-sectional and prospective design, and the long-term follow-up.

## Conclusion

In conclusion, assessing sUmod additionally to creatinine or cystatin C allows a more precise risk modeling for all-cause and cardiovascular mortality and might potentially improve risk stratification. Patients in whom only eGFR is decreased but sUmod remains high are more likely to be male and suffering from heart failure, possibly due to a sUmod mediated enhanced tubular activation of the NKCL-cotransporter. In patients in whom only sUmod is decreased but eGFR remains high we observed a higher prevalence of CAD, a higher fatty liver index and elevated triglycerides that might point to metabolic disturbances as key drivers of increased mortality. The joint consideration of eGFR and uromodulin may have the potential to dissect different forms of cardiorenal syndromes according to their main pathophysiological drivers. Distinct intrinsic metabolic and congruent clinical patterns related to sUmod/eGFR profiles as addressed here for the first time may also govern the susceptibility to risk indicators for kidney health and disease.

## Data Availability Statement

The data analyzed in this study is subject to the following licenses/restrictions: Due to the articles of Ludwigshafen Risk and Cardiovascular Health (LURIC) Study gGmbH, which needs to acknowledge the German Data Protection Act and the consent given by the study participants, data cannot be released to the public domain. The exploitation of the (LURIC) Study database is governed by the articles of the LURIC Study GmbH (non-profit LLC), registered under number HRB 7668 at the commercial registry of Freiburg in Breisgau, Germany. According to the articles of the organization, data access is made available to researchers upon request and approval. This procedure makes sure that rules of good scientific practice are followed and that credit is given to the people who have been in charge of the design and the organization of the study. Interested researchers are invited to address their request or proposal to Kai Grunwald (Kai.Grunwald@weitnauer.net) or to the Principal Investigator of the LURIC Study, Winfried März (winfried.maerz@luric-online.de) who are in charge of supervising ethical and legal aspects of the LURIC study. Finally, the authors confirm that they accessed these data upon approval by LURIC and that all other researchers can access the data upon approval in the same manner the authors did. Similarly, the data from VIVIT cannot be released to the public domain due to Austrian Data Protection Act, but access is also made available to researchers upon request and approval (Contact: Heinz Drexel; vivit@lkhf.at). Requests to access these datasets should be directed to Heinz Drexel; vivit@lkhf.at, winfried.maerz@luric-online.de.

## Ethics Statement

The studies involving human participants were reviewed and approved by the ethics committee of the Landesärztekammer Rheinland-Pfalz [LURIC, #837.255.97(1394)] and of Vorarlberg (VIVIT, EK-2-2013/0008). The patients/participants provided their written informed consent to participate in this study.

## Author Contributions

GD, MK, BY, and AL designed and performed research, made statistical analyses, and drafted the manuscript. BK, WM, and HD designed research and corrected the manuscript. JS drafted the manuscript. HS measured serum uromodulin and corrected the manuscript. All authors contributed to the article and approved the submitted version.

## Funding

LURIC was supported by the 7th Framework Program RiskyCAD (grant agreement number 305739) of the European Union and the H2020 Program TO_AITION (grant agreement number 848146) of the European Union. The work of WM and MK was supported as part of the Competence Cluster of Nutrition and Cardiovascular Health (nutriCARD) which was funded by the German Federal Ministry of Education and Research. The work of GD was supported by the European Union's Horizon 2020 research and innovation programme under the ERA-Net Cofund action N° 727565 (OCTOPUS project) and the German Ministry of Education and Research (grant number 01EA1801A). The funding sources were not involved in study design, in the collection, analysis and interpretation of data, in the writing of the report, and in the decision to submit the article for publication.

## Conflict of Interest

WM reports grants and personal fees from AMGEN, BASF, Sanofi, Siemens Diagnostics, Aegerion Pharmaceuticals, Astrazeneca, Danone Research, Numares, Pfizer, Hoffmann LaRoche: personal fees from MSD, Alexion; grants from Abbott Diagnostics, all outside the submitted work. WM and MK are employed with Synlab Holding Deutschland GmbH. JS declares a patent at the University Charite, Berlin pending, and no other conflict of interest. BK reports travel support a/o lecture fees a/o advisory board memberships from Astellas, Bayer, BMS, Boehringer Ingelheim, Chiesi, Hexal, Pfizer, Sanofi, Servier, Vifor all outside the submitted work. MK reports lecture fees from Bayer outside the submitted work. The remaining authors declare that the research was conducted in the absence of any commercial or financial relationships that could be construed as a potential conflict of interest.

## Publisher's Note

All claims expressed in this article are solely those of the authors and do not necessarily represent those of their affiliated organizations, or those of the publisher, the editors and the reviewers. Any product that may be evaluated in this article, or claim that may be made by its manufacturer, is not guaranteed or endorsed by the publisher.

## References

[1] Kottgen A Glazer NL Dehghan A Hwang SJ Katz R Li M. Multiple loci associated with indices of renal function and chronic kidney disease. Nat Genet. (2009) 41:712–7. 10.1038/ng.377 19430482 PMC3039280

[2] Kottgen A Hwang SJ Larson MG Van Eyk JE Fu Q Benjamin EJ. Uromodulin levels associate with a common UMOD variant and risk for incident CKD. J Am Soc Nephrol. (2010) 21:337–44. 10.1681/ASN.2009070725 19959715 PMC2834540

[3] Köttgen A Pattaro C Böger CA Fuchsberger C Olden M Glazer NL. New loci associated with kidney function and chronic kidney disease. Nat Genet. (2010) 42:376–84. 10.1055/s-0030-1266452 20383146 PMC2997674

[4] Padmanabhan S Melander O Johnson T Di Blasio AM Lee WK Gentilini D. Genome-wide association study of blood pressure extremes identifies variant near UMOD associated with hypertension. PLoS Genet. (2010) 6:e1001177. 10.1097/01.hjh.0000378902.13083.3a 21082022 PMC2965757

[5] Ahluwalia TS Lindholm E Groop L Melander O. Uromodulin gene variant is associated with type 2 diabetic nephropathy. J Hypertens. (2011) 29:1731–4. 10.1097/HJH.0b013e328349de25 21738052

[6] Devuyst O Pattaro C. The UMOD locus: insights into the pathogenesis and prognosis of kidney disease. J Am Soc Nephrol. (2018) 29:713–26. 10.1681/ASN.2017070716 29180396 PMC5827601

[7] Srivastava R Micanovic R El-Achkar TM Janga SC. An intricate network of conserved DNA upstream motifs and associated transcription factors regulate the expression of uromodulin gene. J Urol. (2014) 192:981–9. 10.1016/j.juro.2014.02.095 24594405

[8] Schaeffer C Devuyst O Rampoldi L. Uromodulin: roles in health and disease. Annu Rev Physiol. (2021) 83:477–501. 10.1146/annurev-physiol-031620-092817 33566673

[9] Micanovic R Chitteti BR Dagher PC Srour EF Khan S Hato T. Tamm-Horsfall protein regulates granulopoiesis and systemic neutrophil homeostasis. J Am Soc Nephrol. (2015) 26:2172–82. 10.1681/ASN.2014070664 25556169 PMC4552115

[10] Micanovic R LaFavers K Garimella PS Wu XR El-Achkar TM. Uromodulin (Tamm-Horsfall protein): guardian of urinary and systemic homeostasis. Nephrol Dial Transplant. (2019) 35:33–43. 10.1093/ndt/gfy394 30649494 PMC8205501

[11] Delgado GE Kleber ME Scharnagl H Kramer BK Marz W Scherberich JE. Serum uromodulin and mortality risk in patients undergoing coronary angiography. J Am Soc Nephrol. (2017) 28:2201–10. 10.1681/ASN.2016111162 28242751 PMC5491294

[12] Leiherer A Muendlein A Saely CH Ebner J Brandtner EM Fraunberger P. Serum uromodulin is a predictive biomarker for cardiovascular events and overall mortality in coronary patients. Int J Cardiol. (2017) 231:6–12. 10.1016/j.ijcard.2016.12.183 28089453

[13] Steubl D Buzkova P Garimella PS Ix JH Devarajan P Bennett MR. Association of serum uromodulin with mortality and cardiovascular disease in the elderly-the Cardiovascular Health Study. Nephrol Dial Transplant. (2019) 35:1399–405. 10.1093/ndt/gfz008 30903163 PMC7462724

[14] Garimella PS Lee AK Ambrosius WT Bhatt U Cheung AK Chonchol M. Markers of kidney tubule function and risk of cardiovascular disease events and mortality in the SPRINT trial. Eur Heart J. (2019) 40:3486–93. 10.1093/eurheartj/ehz392 31257404 PMC6837159

[15] Then C Then HL Lechner A Thorand B Meisinger C Heier M. Serum uromodulin and risk for cardiovascular morbidity and mortality in the community-based KORA F4 study. Atherosclerosis. (2020) 297:1–7. 10.1016/j.atherosclerosis.2020.01.030 32058862

[16] Winkelmann BR Marz W Boehm BO Zotz R Hager J Hellstern P. Rationale and design of the LURIC study–a resource for functional genomics, pharmacogenomics and long-term prognosis of cardiovascular disease. Pharmacogenomics. (2001) 2:S1–73. 10.1517/14622416.2.1.S1 11258203

[17] Leiherer A Muendlein A Kinz E Vonbank A Rein P Fraunberger P. High plasma chemerin is associated with renal dysfunction and predictive for cardiovascular events - insights from phenotype and genotype characterization. Vascul Pharmacol. (2016) 77:60–8. 10.1016/j.vph.2015.08.010 26304698

[18] Rein P Vonbank A Saely CH Beer S Jankovic V Boehnel C. Relation of albuminuria to angiographically determined coronary arterial narrowing in patients with and without type 2 diabetes mellitus and stable or suspected coronary artery disease. Am J Cardiol. (2011) 107:1144–8. 10.1016/j.amjcard.2010.12.011 21324429

[19] R Core Team. R Foundation for Statistical Computing. Vienna (2021).

[20] Pivin E Ponte B de Seigneux S Ackermann D Guessous I Ehret G. Uromodulin and nephron mass. Clin J Am Soc Nephrol. (2018) 13:1556–7. 10.2215/CJN.03600318 30054352 PMC6218822

[21] Pruijm M Ponte B Ackermann D Paccaud F Guessous I Ehret G. Associations of urinary uromodulin with clinical characteristics and markers of tubular function in the general population. Clin J Am Soc Nephrol. (2016) 11:70–80. 10.2215/CJN.04230415 26683888 PMC4702229

[22] Devuyst O Olinger E Rampoldi L. Uromodulin: from physiology to rare and complex kidney disorders. Nat Rev Nephrol. (2017) 13:525–44. 10.1038/nrneph.2017.101 28781372

[23] Rampoldi L Scolari F Amoroso A Ghiggeri G Devuyst O. The rediscovery of uromodulin (Tamm-Horsfall protein): from tubulointerstitial nephropathy to chronic kidney disease. Kidney Int. (2011) 80:338–47. 10.1038/ki.2011.134 21654721

[24] Zhou J Chen Y Liu Y Shi S Wang S Li X. Urinary uromodulin excretion predicts progression of chronic kidney disease resulting from IgA nephropathy. PLoS ONE. (2013) 8:e71023. 10.1371/journal.pone.0071023 23990922 PMC3750049

[25] Zylka A Dumnicka P Kusnierz-Cabala B Gala-Bladzinska A Ceranowicz P Kucharz J. Markers of glomerular and tubular damage in the early stage of kidney disease in type 2 diabetic patients. Mediators Inflamm. (2018) 2018:7659243. 10.1155/2018/7659243 30158836 PMC6109534

[26] Bullen AL Katz R Lee AK Anderson CAM Cheung AK Garimella PS. The SPRINT trial suggests that markers of tubule cell function in the urine associate with risk of subsequent acute kidney injury while injury markers elevate after the injury. Kidney Int. (2019) 96:470–9. 10.1016/j.kint.2019.03.024 31262489 PMC6650383

[27] Enko D Meinitzer A Scherberich JE März W Herrmann M Artinger K. Individual uromodulin serum concentration is independent of glomerular filtration rate in healthy kidney donors. Clin Chem Lab Med. (2020) 59:563–70. 10.1515/cclm-2020-0894 33048833

[28] Algharably EAH Bolbrinker J Lezius S Reibis R Wegscheider K Voller H. Uromodulin associates with cardiorenal function in patients with hypertension and cardiovascular disease. J Hypertens. (2017) 35:2053–8. 10.1097/HJH.0000000000001432 28598953

[29] Mutig K Kahl T Saritas T Godes M Persson P Bates J. Activation of the bumetanide-sensitive Na+,K+,2Cl- cotransporter (NKCC2) is facilitated by Tamm-Horsfall protein in a chloride-sensitive manner. J Biol Chem. (2011) 286:30200–10. 10.1074/jbc.M111.222968 21737451 PMC3191059

[30] Trudu M Janas S Lanzani C Debaix H Schaeffer C Ikehata M. Common noncoding UMOD gene variants induce salt-sensitive hypertension and kidney damage by increasing uromodulin expression. Nat Med. (2013) 19:1655–60. 10.1038/nm.3384 24185693 PMC3856354

[31] Welker P Böhlick A Mutig K Salanova M Kahl T Schlüter H. Renal Na+-K+-Cl- cotransporter activity and vasopressin-induced trafficking are lipid raft-dependent. Am J Physiol Renal Physiol. (2008) 295:F789–802. 10.1152/ajprenal.90227.2008 18579701 PMC2536870

[32] Olinger E Lake J Sheehan S Schiano G Takata T Tokonami N. Hepsin-mediated processing of uromodulin is crucial for salt-sensitivity and thick ascending limb homeostasis. Sci Rep. (2019) 9:12287. 10.1038/s41598-019-48300-3 31444371 PMC6707305

[33] Graham LA Aman A Campbell DD Augley J Graham D McBride MW. Salt stress in the renal tubules is linked to TAL-specific expression of uromodulin and an upregulation of heat shock genes. Physiol Genom. (2018) 50:964–72. 10.1152/physiolgenomics.00057.2018 30216136 PMC6293113

[34] Nie M Bal MS Liu J Yang Z Rivera C Wu XR. Uromodulin regulates renal magnesium homeostasis through the ion channel transient receptor potential melastatin 6 (TRPM6). J Biol Chem. (2018) 293:16488–502. 10.1074/jbc.RA118.003950 30139743 PMC6200953

[35] Tokonami N Takata T Beyeler J Ehrbar I Yoshifuji A Christensen EI. Uromodulin is expressed in the distal convoluted tubule, where it is critical for regulation of the sodium chloride cotransporter NCC. Kidney Int. (2018) 94:701–15. 10.1016/j.kint.2018.04.021 30007527

[36] Leiherer A Muendlein A Saely CH Brandtner EM Geiger K Fraunberger P. The value of uromodulin as a new serum marker to predict decline in renal function. J Hypertens. (2018) 36:110–8. 10.1097/HJH.0000000000001527 28858977

[37] Thornley C Dawnay A Cattell WR. Human Tamm-Horsfall glycoprotein: urinary and plasma levels in normal subjects and patients with renal disease determined by a fully validated radioimmunoassay. Clin Sci. (1985) 68:529–35. 10.1042/cs0680529 3979015

[38] Scherberich JE Wolf G Albers C Nowack A Stuckhardt C Schoeppe W. Glomerular and tubular membrane antigens reflecting cellular adaptation in human renal failure. Kidney Int Suppl. (1989) 27:S38–51. 2636672

[39] Steubl D Block M Herbst V Nockher WA Schlumberger W Satanovskij R. Plasma uromodulin correlates with kidney function and identifies early stages in chronic kidney disease patients. Medicine. (2016) 95:e3011. 10.1097/MD.0000000000003011 26962815 PMC4998896

[40] Afsar B Ortiz A Covic A Solak Y Goldsmith D Kanbay M. Focus on renal congestion in heart failure. Clin Kidney J. (2016) 9:39–47. 10.1093/ckj/sfv124 26798459 PMC4720202

[41] Blankstein R Bakris GL. Renal hemodynamic changes in heart failure. Heart Fail Clin. (2008) 4:411–23. 10.1016/j.hfc.2008.03.006 18760753

[42] Alesutan I Luong TTD Schelski N Masyout J Hille S Schneider MP. Circulating uromodulin inhibits vascular calcification by interfering with pro-inflammatory cytokine signaling. Cardiovasc Res. (2020) 117:930–41. 10.1093/cvr/cvaa081 32243494

