# Supplementary material for: Combined Use of Serum Uromodulin and eGFR to Estimate Mortality Risk
Source: Front Med (Lausanne). 2021 Sep 8;8:723546. doi: 10.3389/fmed.2021.723546 (PMC8455921; doi:10.3389/fmed.2021.723546)
Supplement: Supplementary file 1 [file Data_Sheet_1.PDF]

frontiers

Supplementary Material

1 Supplementary Data

1.1 Laboratory procedures

Fasting blood samples were obtained by venipuncture at each study entry. A detailed summary of analytic methods has been reported previously [1, 2]. High-sensitive C-reactive protein (CRP) and cystatin C were measured by immunonephelometry (N-High-Sensitive CRP; N-Latex Cystatin C, Dade Behring, Marburg, Germany) using a Behring nephelometer II. Serum uromodulin was measured in LURIC using a sensitive ELISA specifically adapted to serum specimens [3, 4] In brief, IgG1 of two affinity purified monoclonal antibodies directed against uromodulin isolated from urine were either used for coating microtiter plates or as detection antibody and processed according to the manufacturer`s manual (Euroimmun Diagnostik, Lübeck, Germany). In VIVIT, uromodulin levels were determined by using ELISA kits (BioVendor, Brno, Czech Republic; catalog no. RD191163200R).

Factor V was measured using plasma deficient for factor V on a STA Stago analyser (Immuno GmbH, Heidelberg, Germany). GOT was measured on a Hitachi 717 analyser using the AST (ASAT/GOT) assay. NT-pro-BNP was measured by electro-chemoluminescence on an Elecsys 2010 (Roche Diagnostics). Renin was determined by RIA using the Active renin kit (Diagnostic Systems Laboratories Deutschland GmbH Sinsheim, Germany) on a Berthold Multi-crystal counter LB2014. Angiotensin II was measured by RIA (LKB Valle 1277, γ Master, Uppsala, Sweden). Catecholamines were measured using the Chromsystems HPLC adrenaline, noradrenaline/Waters Millennium chromatography with Waters detector 460 (Chromsystems Instruments & Chemicals GmbH, Martinsried, Germany).

1.2 Definition of clinical variables and endpoints

The presence of a visible luminal narrowing (>20% stenosis) in at least one of 15 coronary segments was used to define coronary artery disease (CAD) according to the classification of the American Heart Association [1]. Diabetes mellitus was defined according to the 2010 guidelines of the American Diabetes Association as increased fasting (≥126 mg/dl) and/or post-challenge (2 h after a 75 g glucose

load >200 mg/dl) glucose and/or elevated glycated hemoglobin (>6.5%) and/or history of diabetes. Hypertension was defined as a systolic and/or diastolic blood pressure ≥140 and/or ≥90 mm Hg or a history of hypertension. The glomerular filtration rate was estimated by using the 2012 CKD-EPI eGFRcreat-cys equation [5]. For 3051 study participants in LURIC and 529 in VIVIT information on both sUmod and CKD-EPI eGFRcreat-cys was available and those comprised the sample for further analyses. The fatty liver index was calculated as described by Bedogni et al. [6].

Information on vital status was obtained from local registries. Death certificates, medical records of local hospitals, and autopsy data were reviewed independently by two experienced clinicians who were blinded to patient characteristics and who classified the causes of death. In cases of disagreement or uncertainty concerning the coding of a specific cause of death the decision was made by a principal investigator (W.M).

2 References

1. Winkelmann BR, Marz W, Boehm BO*, et al.* Rationale and design of the LURIC study--a resource for functional genomics, pharmacogenomics and long-term prognosis of cardiovascular disease. *Pharmacogenomics* 2001; **2:** S1-73.

2. Rein P, Vonbank A, Saely CH*, et al.* Relation of albuminuria to angiographically determined coronary arterial narrowing in patients with and without type 2 diabetes mellitus and stable or suspected coronary artery disease. *The American journal of cardiology* 2011; **107:** 1144-1148.

3. Steubl D, Block M, Herbst V*, et al.* Plasma Uromodulin Correlates With Kidney Function and Identifies Early Stages in Chronic Kidney Disease Patients. *Medicine* 2016; **95:** e3011.

4. Scherberich JE, Gruber R, Nockher WA*, et al.* Serum uromodulin-a marker of kidney function and renal parenchymal integrity. *Nephrology, dialysis, transplantation : official publication of the European Dialysis and Transplant Association - European Renal Association* 2018; **33:** 284-295.

5. Inker LA, Schmid CH, Tighiouart H*, et al.* Estimating glomerular filtration rate from serum creatinine and cystatin C. *The New England journal of medicine* 2012; **367:** 20-29.

6. Bedogni G, Bellentani S, Miglioli L*, et al.* The Fatty Liver Index: a simple and accurate predictor of hepatic steatosis in the general population. *BMC gastroenterology* 2006; **6:** 33.

3 Supplementary Figures and Tables

3.1 Supplementary Figures

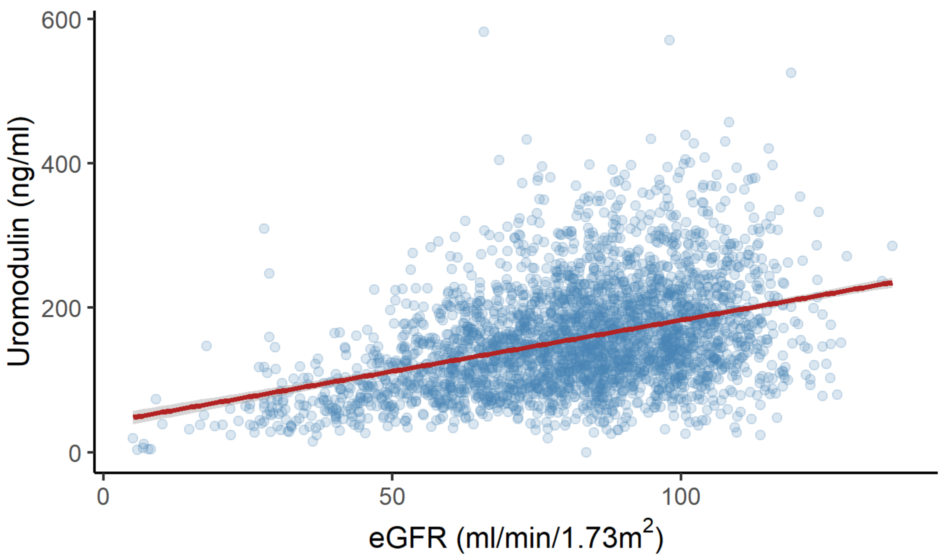

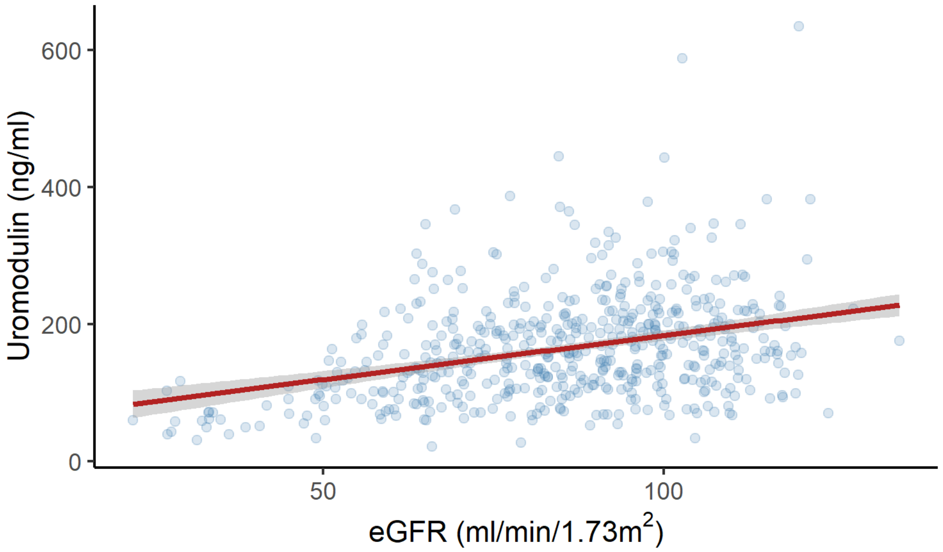

Supplementary Figure 1: Correlation between sUmod and eGFR in LURIC (upper) and VIVIT (lower). The Spearman correlation coefficient is 0.374 (p <0.001) and 0.327 (p <0.001) respectively.

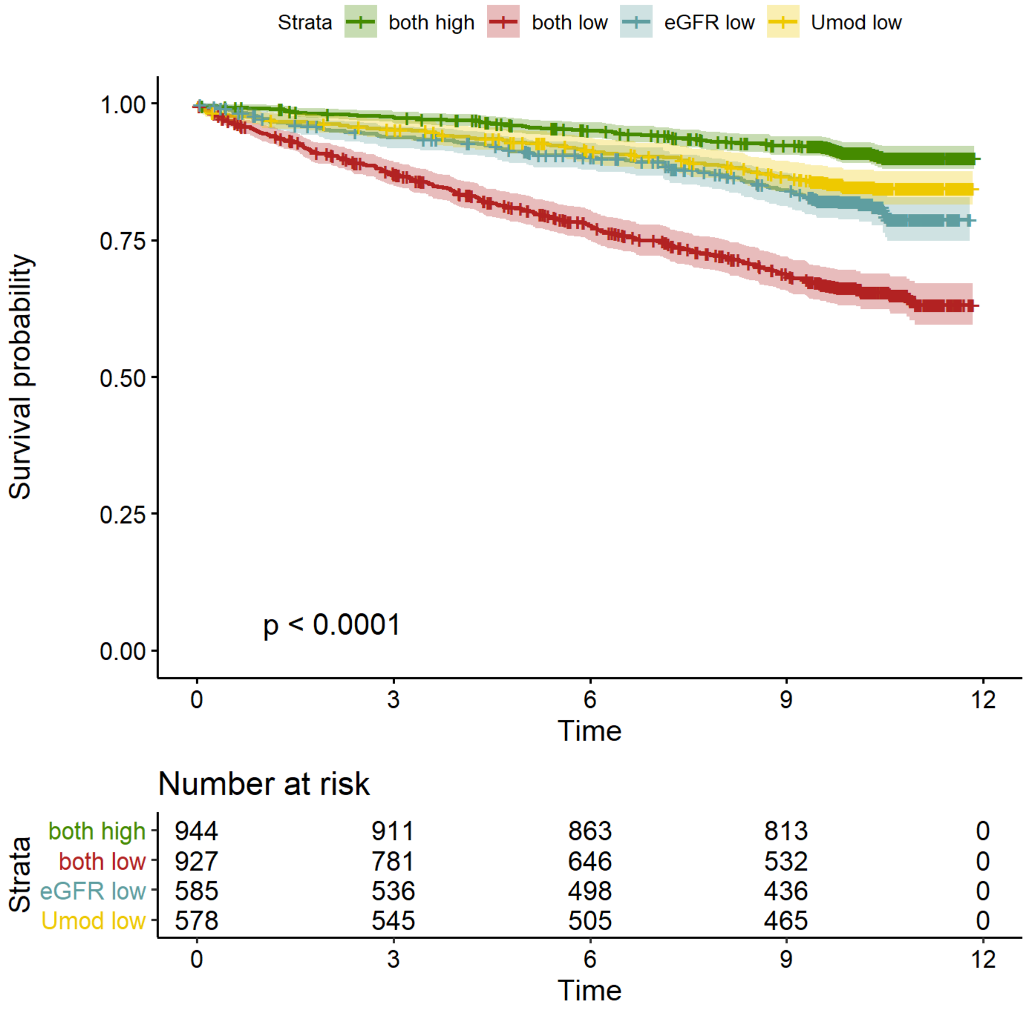

Supplementary Figure 2. Cardiovascular mortality according to eGFR/sUmod groups. The thresholds chosen to separate the patients were the median values of uromodulin (146 ng/ml) and eGFR (84 ml/min/1.73m^2) in LURIC; 95% confidence intervals are shown as ribbons.

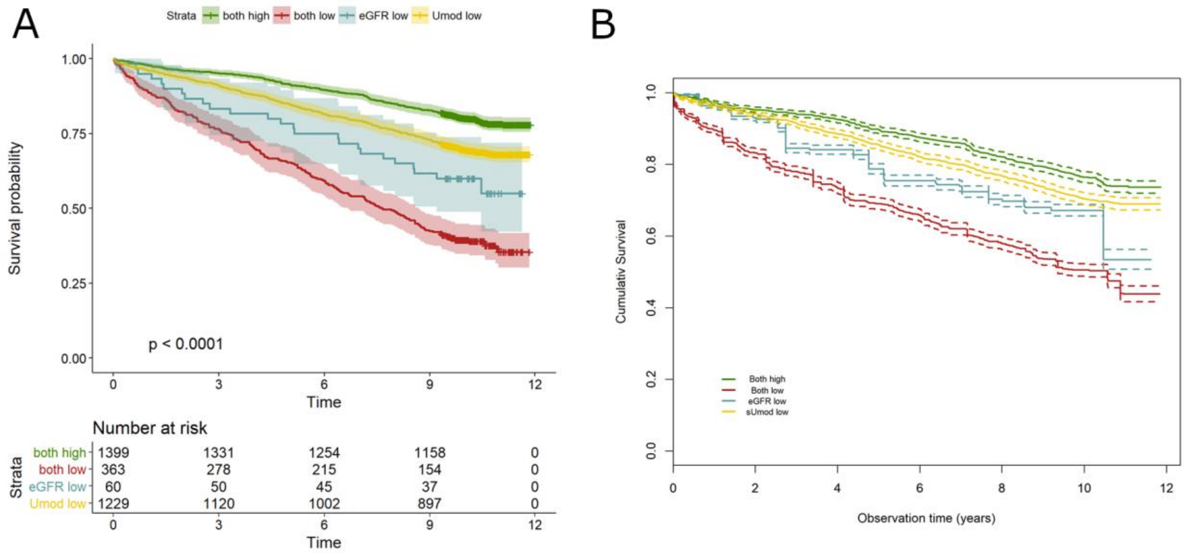

Supplementary Figure 3. Survival according to eGFR/sUmod groups. Kaplan-Meier survival curves with 95% confidence intervals as ribbons (A) and adjusted survival curves with eGFR/ sUmod groups balanced for age, sex, LDL-C, HDL-C, smoking, hypertension and diabetes mellitus by inverse variance weighting (B) in LURIC. The P-value of the robust score test was <0.001; 95% confidence intervals are shown as dashed lines. The thresholds chosen to separate the patients were 150 ng/ml for sUmod and 60 ml/min/1.73m^2 for eGFR; 95% confidence intervals are shown as ribbons.

3.2 Supplementary Tables

Supplementary Table 1: Association of sUmod-eGFR groups with all-cause mortality

		eGFR <> 84 ml/min/1.73m^2 sUmod <>146 ng/ml		eGFR <> 60 ml/min/1.73m^2 sUmod <>150 ng/ml	
sUmod	eGFR	HR (95% CI)	P*	HR (95% CI)	P*
high	high	1		1	
high	low	1.43 (1.13-1.81)	0.002	1.68 (0.77-3.66)	0.193
low	high	1.32 (1.03-1.69)	0.028	1.28 (1.09-1.49)	0.002
low	low	2.03 (1.63-2.52)	<0.001	2.69 (2.01-3.60)	<0.001

* P-value of the robust score of adjusted survival curves with eGFR/ sUmod groups balanced for age, sex, BMI, LDL-C, HDL-C, coronary artery disease, smoking, hypertension and diabetes mellitus by inverse variance weighting in LURIC

Supplementary Table 2. LURIC study characteristics according to serum uromodulin (<150 or ≥150 ng/mL) and eGFR (<60 or ≥60 mL/min/1.73 m^2) groups

Variable	both high	both low	sUmod high, eGFR low	sUmod low, eGFR high	P ANOVA	P Posthoc		
	**UHGH**	**ULGL**	**UHGL**	**ULGH**		**UHGH vs UHGL**	**UHGH vs ULGH**	**UHGL vs ULGH**
N	1399	363	60	1229				
Age (years)	60(10)	71(8.4)	71(7.7)	63(10)	<0.001	<0.001	<0.001	<0.001
Male sex (%)	69	61	47	75	<0.001	0.001	0.002	<0.001
BMI (kg/m^2)	27(4)	28(4)	27(5)	28(4)	0.037	0.995	0.017	0.922
LDL-C (mg/dl)	119(34.9)	111(34.6)	112(30.1)	116(33.3)	0.001	0.370	0.065	0.816
HDL-C (mg/dl)	40(10.8)	35.8(10.2)	40.1(13.3)	37.9(10.7)	<0.001	1.000	<0.001	0.418
TG (mg/dl)	139(104-188)	164(124-216)	142(101-199)	150(113-209)	<0.001	1.000	<0.001	0.558
systolic BP (mmHg)	139(22.9)	146(25.8)	142(23.8)	142(23.3)	<0.001	0.718	0.009	0.999
diastolic BP (mmHg)	81.2(11.3)	79.3(12)	79.5(12.5)	81.3(11.1)	0.013	0.649	0.999	0.627
Magnesium (mmol/l)	0.85(0.0898)	0.887(0.12)	0.896(0.126)	0.841(0.0886)	<0.001	0.001	0.110	<0.001
Fasting glucose (mg/dl)	100(92.6-113)	106(95.7-128)	100(93.2-112)	104(94.7-124)	<0.001	0.999	<0.001	0.325
hsCRP (mg/l)	2.54(1.1-6.77)	6.82(2.81-14.2)	5.5(2.1-15.2)	3.74(1.38-9.04)	<0.001	0.002	<0.001	0.230
NT-proBNP (ng/ml)	210(85-551)	1210(492-2860)	662(364-2440)	281(108-801)	<0.001	<0.001	<0.001	<0.001
Renin (pg/ml)	17(9-36)	32.5(14-89.8)	23(12-60.8)	19(10-39)	<0.001	0.007	0.050	0.069

Angiotensin II (ng/L)	20(12-34)	22(14-42)	27.5(15-49.5)	19(12-32.5)	<0.001	0.004	0.980	0.003
Albumin (g/dl)	4.45(0.525)	4.25(0.545)	4.41(0.687)	4.34(0.564)	<0.001	0.956	<0.001	0.808
GOT (U/l)	12.1(8.71)	11.7(7.54)	12.1(7.49)	11.7(6.94)	0.705	1.000	0.693	0.981
FV (U/dl)	113(20.7)	112(23.4)	119(20.8)	114(22.2)	0.259	0.362	0.592	0.579
Fatty liver index	49.5(26.9-72.3)	57.4(36.5-77)	52.6(23.2-73.5)	57.4(34.2-77.8)	<0.001	0.999	<0.001	0.332
eGFR (ml/min/1.73 m^2)	90(14)	45(12)	53(7)	85(15)	<0.001	<0.001	<0.001	<0.001
Uromodulin (ng/ml)	219(59.7)	83.1(33.1)	191(37.6)	108(28.9)	<0.001	<0.001	<0.001	<0.001
CystatinC (mg/l)	0.85(0.77-0.95)	1.45(1.29-1.74)	1.27(1.19-1.35)	0.92(0.82-1.03)	<0.001	<0.001	<0.001	<0.001
Diabetes mellitus (%)	32	57	48	43	<0.001	0.017	<0.001	0.505
Coronary artery disease (%)	73	85	75	82	<0.001	0.882	<0.001	0.281
Heart failure (%)	26	55	55	34	<0.001	<0.001	<0.001	0.002
Hypertension (%)	67	84	87	75	<0.001	0.002	<0.001	0.039
Smoking (active/ex/never, %)	24/38/38	14/45/41	8/47/45	25/44/31	<0.001	0.012	0.001	0.004
